# SG-APSIC1094: Impact of environmental pollution on skin antimicrobial peptide genes expression revealed by transcriptome profiling

**DOI:** 10.1017/ash.2023.42

**Published:** 2023-03-16

**Authors:** Xuelan Gu, Xiao Cui, Hong Zhang, Grace Mi

**Affiliations:** Unilever, China; Unilever Research and Development Center, Shanghai, China; Unilever Research and Development Center, Shanghai, China; Unilever Research and Development Center, Shanghai, China

## Abstract

**Objectives:** Pollution exposure is associated with several dermatological conditions including acne, atopic dermatitis, and psoriasis. Antimicrobial peptides (AMPs) are key effectors of innate defense, and some AMPs are involved in inflammatory skin conditions. In this study, we aimed to characterize expression changes of human AMPs under different in-vitro pollution exposures. **Methods:** RNA-seq profiling was conducted on normal human primary epidermal keratinocytes (NHEK) treated with either a vehicle control, or benzo[a]pyrene (BaP) and on pigmented living skin equivalent models (pLSE) treated with either a vehicle control, ozone, or vehicle exhaust. Differential expressed genes (DEGs) were identified with R scripts. DEGs of PM2.5 were obtained from the literature and the GEO database. Also, 180 human AMP genes were obtained from a UDAMP database. UpSetPlot was used to plot DEGs overlaps. MetaVolcano was used to identify frequently changed AMPs. **Results:** We used in-house and published transcriptome profiles to identify AMP genes that displayed altered expression under in-vitro pollution exposure. Of the 180 AMP genes under investigation, 37 showed significant changes in expression in at least 1 of the 5 experiments. Using MetaVolcano, 13 AMP genes were identified to be frequently and consistently changed. Several AMPs associated with inflammation and skin diseases were frequently upregulated, including S100A8, S100A9, LCN2, HBD3, RNASE7, and CXCL1. Only 3 frequently downregulated AMP genes were identified, including CXCL14, which is reported to be a noninflammatory AMP that is highly expressed in healthy skin and is downregulated in skin diseases. **Conclusions:** The data sets suggest that expression of both proinflammatory and homeostatic AMPs can be perturbed by pollution. These findings provide new clues to explain how pollution affects skin innate defense, host–microbe interactions and contributes to abnormal skin conditions. Normalizing aberrant AMP expression may be a potential approach to treat pollution associated skin disorders in the future.

